# Hide Bound

**Published:** 1887-06

**Authors:** 


### HIDE-BOUND.

It is remarkable that so few homœopaths seem to understand the true nature of the law of similars. Thus we find this law often spoken of as if it were one of man's dicta, to be changed at pleasure. The law of similars *must* be a law of nature or it is worthless. Nature's laws are as eternal as nature itself; they cannot change. Our knowledge of them, and our ability to use them, should continually change and improve; and it must be confessed that our knowledge of and our ability to apply this therapeutic law is almost in its infancy at present. Therefore let us develop this law, rather than desert it for each fanciful theory which man's imagination cojures up.

These thoughts have been suggested by the following sentence from a note sent the writer by Dr. S. Swan: "You *must* remember that all the members [of the I. H. A.] are not as hide-bound as you are. They *must* grow out of old ideas as the new ones are proved true. I expect to shock your sensibilities more."

We presume the "old ideas" here referred to are the law of similars, and by the "new ones" Dr. S. refers to his "generalization."

As this generalization is at present, being unproven, simply the dictum of Dr. Swan (which, indeed, he has materially changed in the last few years) we will be pardoned for being "hide-bound."

Some years ago one of Dr. Swan's ardent admirers sent the writer an indignant letter for opposing Dr. Swan's theories, saying: "You are wrong; I make some wonderful cures with

Dr. Swan's high and highest potencies; we make cures first and provings afterward"!! As this method of studying therapeutics is a reversal of "Hahnemann's strict inductive method," we may again be pardoned for being so hide-bound as to discredit it. Dr. Swan's therapeutics being in every way at variance with "Hahnemann's strict inductive method," we ask once more, Which shall it be—Hahnemann or Swan, Homœopathy or empiricism? The I. H. A. cannot advocate both.

